# Farm animal genetic resources and the COVID-19 pandemic

**DOI:** 10.1093/af/vfaa049

**Published:** 2021-02-05

**Authors:** Gustavo Gandini, Sipke Joost Hiemstra

**Affiliations:** 1 Department DIMEVET, University of Milan, Lodi, Italy; 2 Centre for Genetic Resources, Wageningen University & Research, AH Wageningen, The Netherlands

**Keywords:** agroecology, COVID-19, farm animal genetic resources, local breeds, typical food product

**Implications**

- Endangered breeds in fragile socio-economic contexts are particularly exposed to population decline and stochastic perturbations expected following COVID-19 pandemic.
- The interest of richer societies for short food chains, natural food and traceability of products can create opportunities for local breeds production systems in the context of COVID-19.
- National surveys on the breed diversity status following COVID-19 should address negative situations and opportunities.
- COVID-19 has revealed risks, fragilities and inequalities for our food systems. The aim is to build resilience at all levels. Agroecology, which implies to conserve breed diversity, can play an important role.

Since early 2020, the COVID-19 pandemic is deeply affecting human health and is causing a severe worldwide economic and social crisis. The real dimensions of the impacts are yet to be determined and will differ among geographical areas and economic sectors, including agriculture and animal production. Effects are expected to be higher in poorer and more vulnerable countries and population strata.

Farm animal genetic resources (AnGR) refer to the diversity present within and between farm animal species, the diversity that is necessary for facing the current challenges of food production in agriculture and to meet future demands, in the scenario of the ongoing growth of the human population. A specific focus in AnGR management is on the genetic diversity between and within local breeds and on how to stop the erosion that started in the middle of the last century, with intensification and specialization of agriculture. This note proposes some considerations on the possible effects of the COVID-19 crisis on local breed diversity conservation and mitigation measures.

It has been shown that the DNA sequence of the SARS-CoV-2 virus, the agent of the COVID-19 disease, has similarities with other coronaviruses infecting bats, suggesting that this mammal was the original animal reservoir. The next step, still unclear, is that the virus has arrived in humans through an intermediate animal host, the Chinese pangolin (*Manis pentadactyla)*. Given this framework, it should be noted that much attention has been given to the possible interface between humans and the pangolin, the Chinese “wet markets”, and that much lesser consideration is addressed to the wider interface between humans and the bats’ forest ecosystems. The density of contacts between human populations and previously undisturbed forests is recognized to increase the risk of spillovers. This has been documented for HIV, Ebola virus, and other pathogens (Quammen, 2012).

Some features of animal production systems in different areas of the world contribute to enhancing human–wildlife interactions by clearing new forest areas and by pushing smallholding farmers toward the remaining forests. Although the rate of global net forest loss has somehow decreased in this century, in the period 2010–2015, an average of 3.3 million ha of forest was still lost each year for the expansion of agricultural land. This inevitably affects the ecology of the forest systems. The entry of humans into areas formerly inhabited by wild animals may provide opportunities for animal viruses to enter the human community. Moreover, intensive farming has been sometimes claimed to be among factors triggering the COVID-19 pandemic, but no evidence has been brought forward in this respect. However, there is a clear link between the growth and intensification of the global livestock sector and the global loss of biodiversity and between livestock production systems in general and other recent zoonotic pandemics, for example, caused by influenza viruses. More intensive livestock systems are considered to be associated with higher zoonotic risks (Wallace, 2016), although a debate is still open about the relationship between the risks and scale/intensity of systems. In the intensive farming context, high animal densities combined with high animal performances and low genetic diversity of the animals, as in intensive poultry and swine production, amplify the risk of emerging and spreading of diseases. Low genetic diversity is associated with the lack of genetic variants providing resistance to the farmed populations against pathogens, with the risk of faster spreading of the disease.

Considering the links between intensive farming and recent pandemics, we should turn this COVID-19 crisis into an opportunity for rethinking animal production including consumers to substantially reduce consumption of animal protein, reconsidering the ecological dimension of animal production systems, aiming for higher standards of “epidemiological sustainability.”

The effects that the COVID-19 pandemic has on the diversity of local breeds, in the short and medium term, are to be determined. Not enough information is yet available. About 30% of the livestock breeds are considered endangered on the basis of their small population sizes (DAD-IS, 2020), although, for 60% of breeds, basic demography is unknown. Besides other threats, endangered breeds are particularly exposed to the effects of population decline and stochastic perturbations that can be expected following COVID-19 pandemic. The weakness of many local breeds, besides their population size, is also often associated with their farming areas or territory, often characterized by particularly fragile socioeconomic contexts, with limited capacity to react to the effects of the pandemic. Emergencies can have a significant effect on farm animal diversity, in particular, because of disruptions to livestock-keeping livelihoods. In addition, emergencies can negatively affect the sustainable management of AnGR.

The restriction of human movements and of people gathering, business closures, and merchandise trade limitations under COVID-19 are proving the vulnerabilities of food chains. Evidence is available for the general livestock farming sector. But how these elements could affect the short food supply chains associated with local breeds is largely unknown. Different pictures are possible. Poor economic farming contexts can be expected to be less affected by the pandemic. Conversely, in richer countries, food chains characterized by local products, gastronomy, and tourism can be expected to be negatively conditioned. However, on the positive side, in such countries, the higher interest of the society for short food chains, natural food, and traceability of products could also create specific opportunities for the local breeds production systems in the context of COVID-19. Some evidence in this direction is observed in Italy, for example, in the Reggiana cattle, a known example of how a typical product can be beneficial to an endangered breed (Gandini et al., 2007). In the 1940s, the population of Reggiana cows was greater than 40,000; however, the number progressively regressed to 550 cows in the early 1980s as Reggiana cows were displaced by Holstein cows with higher milk production. Since 1992, a consortia of dairy producers initiated the production of a branded Parmigiano Reggiano cheese, made exclusively from milk from Reggiana cows. The branded cheese is today sold at premium prices (+45%) compared with standard Parmigiano Reggiano cheese and, since 1992, has been revitalizing the interest in the breed that has gradually increased in cow numbers to 2,700 in 2020. During the COVID-19 quarantine, March–May 2020, direct sales of the branded Parmigiano Reggiano cheese dropped dramatically but, since March, selling via the internet increased consistently compared to previous years. The Historic Rebel cheese, made with local breeds on Valtellina summer pastures, in the Italian Alpine Ark, is showing a similar trend, possibly also linked to the trend toward nature-based tourism (Figure 1). Similarly, in The Netherlands, a clearly positive impact on some short supply chains associated with local breeds is observed. National surveys on the breed diversity status following COVID-19, within the FAO Global Plan of Action for Animal Genetic Resources, should address, identify, and counteract possible negative situations and opportunities.

**Figure 1.**
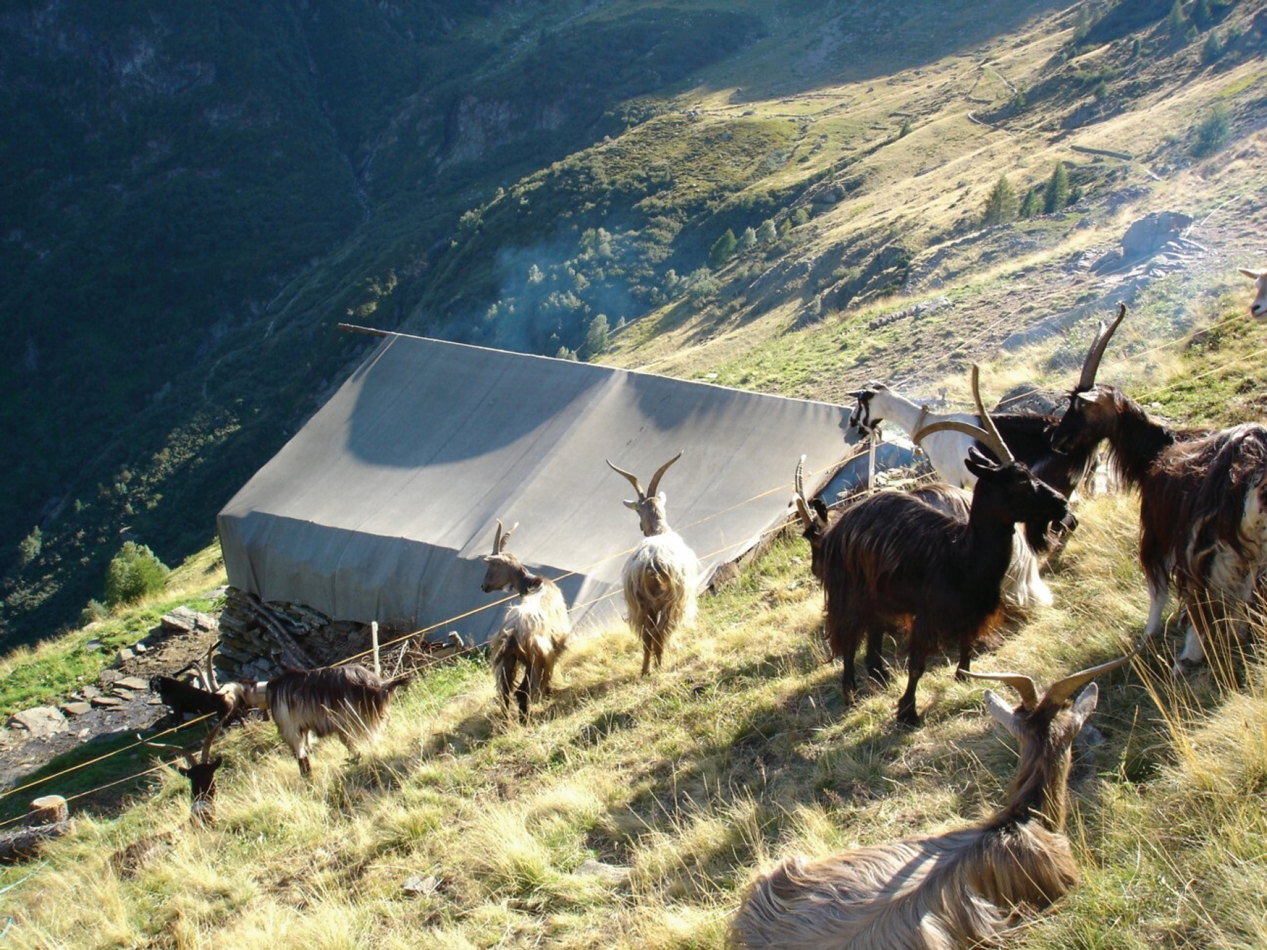
Orobica goats at a farm-based cheese dairy for the Historic Rebel cheese, Italian Alps. How COVID-19 will affect this short food chain?

In general, COVID-19 has revealed risks, fragilities, and inequalities for our food systems. During a severe crisis, the real challenge is to promote system transformations to encounter evolving environmental conditions and human needs, avoiding the return to business as usual. The overall aim is to build resilience in the agriculture sector at all levels. The 2030 Agenda for Sustainable Development underlines the need to take actions directed at transformational change. A transition is needed to more sustainable and resilient food systems capable to produce more, with higher socioeconomic benefits and fewer environmental consequences. In the case of AnGR, for example, a stricter separation between livestock systems and nature areas, sometimes advocated to avoid transmission of zoonosis, might result in negative effects on local breed conservation. On the other hand, the integration of biodiversity and environmental values and other ecosystem services in the development of future livestock systems may provide opportunities for increasing breed diversity.

Agroecology is based on applying ecological concepts and principles to optimize interactions between plants, animals, humans, and the environment while taking into consideration the social aspects that need to be addressed for a sustainable and fair food system. Agroecology can play an important role in building the resilience of our food system that the COVID-19 pandemic has shown to be fragile. Agroecology implies to conserve biodiversity, including breed diversity, and the ecosystem services on which agriculture depends. There is a need for sustainable and feasible policies to conserve and utilize the existing diversity of local breeds in rural development. Agroecology is not a new concept but is gaining more and more interest worldwide, including governments, research, civil society and producer organizations, international institutions, and the private sector. FAO’s engagement is today adding strength to agroecology. Can the agroecological approach be applied on a global scale? Can it respond to the challenge of food security in the world and at the same time contribute to global climate challenges? These are recurrent questions that today receive positive answers from many experts. Obviously, a complex transition period has to be covered. Today agroecology represents a space for challenging our modern food systems where COVID-19 has revealed risks, fragilities, and inequalities.

A final consideration on animal research: COVID-19 sanitary measures are negatively affecting the interactions necessary for research, development, and dissemination through social distancing and loss of employment. The expected postpandemic economic recession might also result in reductions of the financial commitment to animal research by funding agencies, governments, and the private sector. The field of AnGR conservation has generally received limited financial support. On the contrary, the need for higher resilience in agriculture should call for future financial supports to promote diversification in agriculture and livestock production and to promote the use and conservation of farm animal diversity.

*Conflict of interest statement*. None declared.

## About the Authors

**Gustavo Gandini** is professor in animal genetics at the Faculty of Veterinary Medicine, University of Milan. Research interests include management of genetic variation in small livestock populations and conservation of farm animal genetic resources. He was the President of the Working Group on Animal Genetic Resources of EAAP (2002–2009). He is the Dean of the Faculty of Veterinary Medicine of Milan (since 2017) and President of the Ethical Committee of the Faculty (since 2010). His interests include animal bioethics (he is the Animal Protection authority for the Milan Municipality since 2018) and activism in nature conservation (Vice-president of WWF-Italy from 2009 to 2012; since 2000, he works in Gabon for forest conservation).

**Sipke Joost Hiemstra** is Director of the Center for Genetic Resources, the Netherlands (CGN) of Wageningen University and Research, coordinating the Dutch national programme on genetic resources (plants, animals, and forest) for the Dutch Ministry of Agriculture. He is also heading the Animal Genetic Resources group at CGN and is the FAO National Coordinator for Animal Genetic Resources in the Netherlands. CGN has established national gene bank collections for farm animal breeds, initiates scientific research on farm animal genetic diversity, and gives advice to policy makers, breeding associations, breeding industry, and other stakeholders in relation to the conservation and sustainable use of farm animal genetic resources. At the European level, he is chair of the Steering Committee of the European Regional Focal Point for Animal Genetic Resources (ERFP) and initiated the development of the European Network of Animal Gene Banks within the ERFP framework.
